# CONSORT for Reporting Randomized Controlled Trials in Journal and Conference Abstracts: Explanation and Elaboration

**DOI:** 10.1371/journal.pmed.0050020

**Published:** 2008-01-22

**Authors:** Sally Hopewell, Mike Clarke, David Moher, Elizabeth Wager, Philippa Middleton, Douglas G Altman, Kenneth F Schulz, and the CONSORT Group

**Affiliations:** 1 UK Cochrane Centre, Oxford, United Kingdom; 2 Centre for Statistics in Medicine, Wolfson College, Oxford University, Oxford, United Kingdom; 3 School of Nursing and Midwifery, Trinity College Dublin, Dublin, Ireland; 4 Chalmers Research Group, Children's Hospital of Eastern Ontario Research Institute, Ottawa, Canada; 5 Department of Epidemiology and Community Medicine, Faculty of Medicine, University of Ottawa, Ottawa, Canada; 6 Sideview, Princes Risborough, United Kingdom; 7 Discipline of Obstetrics and Gynaecology, The University of Adelaide, Adelaide, Australia; 8 Family Health International, Research Triangle Park, North Carolina, United States of America; University of Bern, Switzerland

## Abstract

**Background:**

Clear, transparent, and sufficiently detailed abstracts of conferences and journal articles related to randomized controlled trials (RCTs) are important, because readers often base their assessment of a trial solely on information in the abstract. Here, we extend the CONSORT (Consolidated Standards of Reporting Trials) Statement to develop a minimum list of essential items, which authors should consider when reporting the results of a RCT in any journal or conference abstract.

**Methods and Findings:**

We generated a list of items from existing quality assessment tools and empirical evidence. A three-round, modified-Delphi process was used to select items. In all, 109 participants were invited to participate in an electronic survey; the response rate was 61%. Survey results were presented at a meeting of the CONSORT Group in Montebello, Canada, January 2007, involving 26 participants, including clinical trialists, statisticians, epidemiologists, and biomedical editors. Checklist items were discussed for eligibility into the final checklist. The checklist was then revised to ensure that it reflected discussions held during and subsequent to the meeting. CONSORT for Abstracts recommends that abstracts relating to RCTs have a structured format. Items should include details of trial objectives; trial design (e.g., method of allocation, blinding/masking); trial participants (i.e., description, numbers randomized, and number analyzed); interventions intended for each randomized group and their impact on primary efficacy outcomes and harms; trial conclusions; trial registration name and number; and source of funding. We recommend the checklist be used in conjunction with this explanatory document, which includes examples of good reporting, rationale, and evidence, when available, for the inclusion of each item.

**Conclusions:**

CONSORT for Abstracts aims to improve reporting of abstracts of RCTs published in journal articles and conference proceedings. It will help authors of abstracts of these trials provide the detail and clarity needed by readers wishing to assess a trial's validity and the applicability of its results.

## Abstract 2

The authors extend the CONSORT Statement to develop a minimum list of essential items to consider when reporting the results of a randomized trial in any journal or conference abstract.

## Introduction

Well-written abstracts of conferences and journal articles reporting randomized controlled trials (RCTs) are important, because readers will often base their initial assessment of a trial on the information reported in an abstract. They may then use this information to decide whether or not to seek more knowledge about the trial, such as by reading the full report if available. In some geographic areas, the abstract of a RCT may be all that health professionals have easy access to, and health-care decisions may be made solely on information reported in it. Where the results of a trial are reported only as a conference abstract, this abstract may provide the only permanent information about a study and the only way that its results can be accessed by most readers [1]. Journal and conference abstracts should contain sufficient information about the trial to serve as an accurate record of its conduct and findings, providing optimal information about the trial within the space constraints of the abstract format. A properly constructed and well-written abstract should also help individuals to assess quickly the validity and applicability of the findings and, in the case of abstracts of journal articles, aid the retrieval of reports from electronic databases [2]. Conference abstracts, in particular, can provide valuable information for systematic reviewers about studies that are not otherwise published, the exclusion of which from the review might introduce bias [3].

### Incomplete and Inaccurate Reporting

A number of studies have highlighted the need for improvements in the reporting of conference abstracts and the abstracts of journal articles presenting the results of RCTs [4]. There are concerns over the accuracy and quality of trial reports published in the proceedings of scientific meetings, including the lack of information about the trial and the robustness of the trial results, compared with results published in a journal article [5–9]. Research has also shown that trial information reported in conference abstracts may differ from that reported in subsequent full publications of the same study [10–13].

The abstract of a journal article has similar limitations to those of an abstract submitted to a scientific meeting. In particular, print space limitations constrain the detail that authors may include on the trial's methodology and results. A journal abstract should be an accurate reflection of what is included in the full journal article and should not include information that does not appear in the body of the paper. Studies comparing the accuracy of information reported in a journal abstract with that reported in the text of the full publication have found claims that are inconsistent with, or missing from, the body of the full article [14–18]. Conversely, omitting important contrary results from the abstract, such as those concerning side effects, could seriously mislead a reader's interpretation of the trial findings [19,20].

### Improving the Reporting of Randomized Trials in Journal and Conference Abstracts

The CONSORT (Consolidated Standards of Reporting Trials) Statement, first published in 1996 [21] and updated in 2001 [22], provides recommendations for reporting RCTs in health-care journals. CONSORT has been endorsed by the World Association of Medical Editors (WAME), the International Committee of Medical Journal Editors (ICMJE), and the Council of Science Editors (CSE). Currently, however, the CONSORT Statement provides limited guidance about preparing abstracts and, while it encourages the use of a structured format, this is not a formal requirement. The ICMJE Uniform Requirements [23] also provide only limited guidance on the format of abstracts for journal articles.

We believe that instructions to authors from journals and conference organizers should provide specific instructions about key elements of a trial that should be reported in an abstract. Indeed, a recent study examining the content of 35 journals' instructions to authors found that only 4% of all words were devoted to the content or format of the abstract [24]. Without a minimum amount of key information on a trial, it is difficult to assess the validity of its results or its applicability.

## Methods

### CONSORT for Abstracts: Development of the Checklist

In collaboration with others in the CONSORT Group, we have extended the current CONSORT Statement to develop a checklist of essential items that authors should consider when reporting the main (i.e., those reporting the pre-specified primary outcome) results of a RCT in any journal or conference abstract.

First, we established a steering committee (MC, SH, DM, PM, and EW). Second, we generated a list of items from existing quality assessment and reporting tools, including the CONSORT Statement [22] and other guidance for the structured reporting of journal abstracts and short reports [25–28]. Third, additional items were generated as part of an empirical study assessing the quality of trials reported in conference proceedings and journal abstracts [29].

We then used a modified Delphi consensus method [30] to select and reduce the number of possible checklist items. A total of 109 participants, who were known to have an interest in the reporting of RCTs, the structure of abstracts, or both were invited (by e-mail) to participate in a Web-based survey and rate the importance of 27 suggested checklist items. The response rate was 61% (*n* = 63) for the first round of the Delphi survey. Respondents included journal editors (13%), health-care professionals (22%), methodologists (40%), statisticians (5%), trialists (7%), and other individuals with expertise in the reporting of RCTs (13%). During three rounds of the survey, participants were asked about their views on the relative importance of the possible checklist items. A more detailed discussion of the Delphi process is included in Text S1.

The results of the survey were presented at a one-day meeting (part of a three-day CONSORT Group meeting) in January 2007, in Montebello, Canada, attended by 26 participants, several of whom also participated in the Delphi survey. The meeting began with a review of the checklist items proposed as a result of the Delphi process. Participants then discussed in small groups whether proposed checklist items should be included, excluded, or modified in the final checklist. These small-group deliberations were further discussed during plenary sessions.

Following the meeting, the checklist was revised and circulated to the steering committee and meeting participants to ensure that it reflected the discussions. The steering committee also developed this explanation and elaboration document, which was circulated through several iterations among the authors.

### CONSORT for Abstracts Checklist: Explanation and Elaboration

We developed this document using the template used to develop the CONSORT and STARD (Standards for Reporting Diagnostic Accuracy) explanatory articles [31,32]. Here each item (see Table 1) is stated, a recent example of good reporting of the item is provided, followed by an explanation that includes the rationale and scientific background and, where possible, discusses the evidence for the item as it relates to a trial reported in a journal or conference abstract.

**Table 1:**
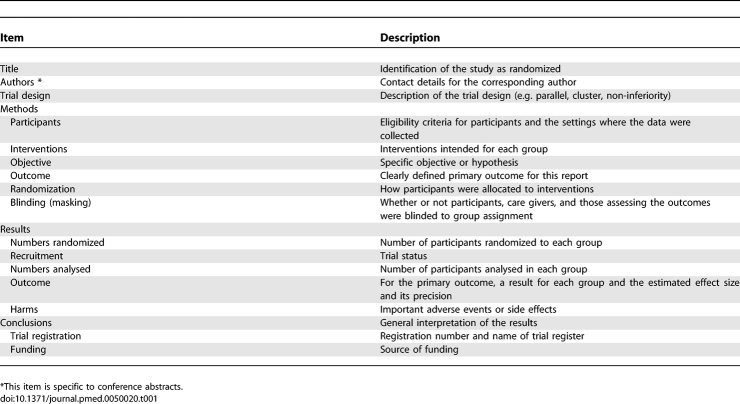
Items to Include when Reporting a Randomized Trial in a Journal or Conference Abstract

## Checklist Items

### TITLE Item: Identification of the study as randomized.

#### Example.

“Effectiveness of a strategy to improve adherence to tuberculosis treatment in a resource poor setting: a cluster randomized trial” [33].

#### Explanation.

The ability to identify a relevant report in an electronic database depends to a large extent on how it was indexed. Indexers may not classify a report as a RCT if the authors do not explicitly report this information [34]. To help ensure that a study is appropriately indexed and identified as a RCT, authors should state explicitly in the title that the participants were randomly assigned to their comparison groups.

### AUTHORS Item: Contact details for the corresponding author.

(This item is specific to conference abstracts)

#### Example.

“Correspondence to: Dr Sally Hopewell, UK Cochrane Centre, Summertown Pavilion, Middle Way, Oxford OX2 7LG, UK. Tel: +44 1865 516300; Fax: +44 1865 516311; Email: *shopewell@cochrane.co.uk*.”

#### Explanation.

Adequate contact details for the corresponding author are particularly important for RCTs reported in conference proceedings. These abstracts may be the only lasting source of information for many trials, as only half of RCTs reported in conference proceedings are subsequently published in full [1]. Adequate contact information would enable readers to contact trialists for additional information or clarifications regarding reported data. Adequate contact details should include the telephone number, postal, and email address of the principal investigator and, if available, the trial Web site.

### TRIAL DESIGN Item: Description of the trial design.

#### Example.

“A cluster randomized controlled trial...” [33].

#### Explanation.

The design of the trial should be described, for example, parallel group, cluster randomized, crossover, factorial, superiority, equivalence or noninferiority, or some other combination of these designs. An important reason for identifying the design of the trial is to ensure appropriate indexing in electronic databases, thus ensuring greater ease of identification [34]. Alerting readers to the design of the trial also provides transparency as to the type of design used to conduct the trial and should reduce the likelihood of inadvertently misinterpreting data. For example, in a report of a cluster trial, readers might misinterpret a small sample size as the number of participants rather than the number of clusters, or vice versa [35].

### METHODS Participants Item: Eligibility criteria for participants and the settings where the data were collected.

#### Example.

“… conducted between June 2003 and January 2005, at 16 government district health centers in Senegal. Patients older than 15 years with newly diagnosed sputum smear-positive pulmonary TB were randomly assigned to the intervention or control group” [33].

#### Explanation.

Every RCT addresses an issue relevant to a particular population or group with the condition of interest. Trialists may further restrict this sample by using eligibility criteria and by performing the trial in a particular setting (for example primary, secondary, or tertiary care). Participant eligibility criteria may relate to demographics, clinical diagnosis, and comorbid conditions. A clear description of the trial participants and setting in which they were studied is needed so that readers may assess the external validity (generalisability) of the trial and determine its applicability to their own setting.

### Interventions Item: Interventions intended for each group.

#### Example.

“Patients were randomized to receive either 100 mg hydrocortisone or matching placebo as follows: the first dose in the evening of the operative day, then 1 dose every 8 hours during the next 3 days. In addition, all patients received oral metoprolol (50–150 mg/d) titrated to heart rate” [36].

#### Explanation.

The essential features of the experimental and comparison interventions should be described. Authors should report details about the interventions, e.g., dose, route of administration, duration of administration, surgical procedure, or manufacturer of inserted device.

### Objective Item: Specific objective or hypothesis.

#### Example.

“To compare the effectiveness of an early switch to oral antibiotics with the standard 7 day course of intravenous antibiotics in severe community acquired pneumonia” [37].

#### Explanation.

The abstract should provide a clear statement of the specific objective or hypothesis addressed in the trial. If more than one objective is addressed, the main objective (i.e., based on the prespecified primary outcome) should be indicated and only key secondary objectives stated [26].

### Outcome Item: Clearly defined primary outcome for this report.

#### Example.

“Main outcome measure: all-cause mortality at 180 days” [38].

#### Explanation.

RCTs assess outcomes for which the interventions are being compared. Most trials have several outcomes, some of which are deemed more important than others. Such rankings are typically reported as primary and secondary outcomes. There is evidence of selective reporting with significant or favourable outcomes being more likely to be published than nonsignificant outcomes [39–41].

Authors should explicitly state the primary outcome for the trial and when it was assessed (e.g., the time frame over which it was measured). The primary outcome is the prespecified outcome considered of greatest importance and is usually the one used in the sample size calculation [22]. In some instances a publication may report an outcome different from the primary outcome. For example, conference abstracts are more likely to report interim analyses than are full publications [8,10], or to present different results for a single trial in a series of abstracts. If the abstract focuses on a secondary outcome of a trial, the abstract should identify both this outcome and the primary outcome of the trial.

### Randomization Item: How participants were allocated to interventions.

#### Example.

“Randomization was computer-generated, with allocation concealment by opaque sequentially numbered sealed envelopes” [42].

#### Explanation.

It is important to conceal the allocation sequence from those assigning participants to the intervention groups. Allocation concealment prevents investigators from influencing which participants are assigned to a given intervention group (i.e., selection bias). Evidence shows that reports of trials reporting inadequate allocation concealment are associated with exaggerated treatment effects [43,44]. Research suggests that adequate allocation concealment is more important in preventing selection bias than are other components of the randomization process, such as the sequence generation (e.g., use of computer or random number table) [45].

Authors should clearly describe the method for assigning participants to interventions. Examples of approaches used to ensure adequate concealment include: centralised (e.g., allocation by a central office) or pharmacy-controlled randomization; sequentially numbered identical containers that are administered serially to participants; on-site computer system combined with allocations kept in a locked, unreadable computer file that investigators can access only after the characteristics of an enrolled participant are entered; and sequentially numbered, opaque sealed envelopes [46].

The method of allocation concealment is generally poorly reported in conference abstracts and in abstracts of journal articles [7,47–49]. For example, in a review of 494 abstracts presented at an oncology conference in 1992 and 2002, only nine (2%) abstracts reported the method of allocation concealment. This information was missing from the remaining 485 conference abstracts, with no improvements seen over the ten-year period [7].

### Blinding (Masking) Item: Whether or not participants, caregivers, and those assessing the outcomes were blinded to group assignment.

#### Example.

“Children, parents, and the research assistants were blinded to group assignment” [50].

#### Explanation.

Blinding refers to the practice of keeping the trial participants, care providers, data collectors, and sometimes those analysing the data, unaware of which intervention is being administered to which participant, so that they will not be influenced by that knowledge. The term masking is sometimes used instead of blinding [51,52] and might be preferable when reporting studies involving eyes and vision. It is important that authors describe whether or not participants, those administering the intervention (usually health-care providers), and those assessing the outcome (the data collectors and analysts) were blinded to the group allocation. Authors should avoid using terms such as “single” or “double” blind as such terms are not well-understood [53].

Information on the method of blinding is poorly reported in conference and journal abstracts [7,8,47–49]. Such reporting is valuable as blinding may be important in protecting against bias [51]. Studies have shown that if investigators are aware of the treatment, their attitudes for or against an intervention can directly affect whether or not they include, or treat, participants in a trial [45]. Furthermore, there is evidence that participants who are aware of their assignment status are more likely to report symptoms, leading to biased results [51]. Perhaps most importantly, if outcome assessors are not blinded to the intervention they are more likely to report favourable outcomes for the intervention which they believe is better [54]. However, unlike allocation concealment, blinding of the participants, health-care providers, and outcome assessors may not always be appropriate or possible, such as in many surgical trials. In this case, authors should report if any form of blinding (such as blinding of data analysts) was used.

### RESULTS Numbers Randomized Item: Number of participants randomized to each group.

#### Example.

“Children (*n* = 633) aged 1–3 randomly allocated to receive fortified milk (*n* = 316) or control milk (*n* = 317)” [55].

#### Explanation.

The number of participants randomized to each intervention group is an essential element of the results of a trial. This number defines the sample size, and readers can use it to assess whether all randomized participants were included in the data analysis. Again, this may be particularly important for conference abstracts reporting interim analyses, if a trial is still open to participant accrual or follow-up [8,10]. Here authors should report the period of recruitment on which the data are based.

### Recruitment Item: Trial status.

#### Example.

“An interim analysis was performed because of slow accrual” [56].

#### Explanation.

Authors should describe the status of the trial and whether it is still ongoing, closed to recruitment, or closed to follow-up. This information is particularly important for conference abstracts, which are more likely than full articles to report interim analyses [10].

If the trial has stopped earlier than planned it is important to say why. Possible reasons for early termination include: slow accrual rates, poor data quality, poor adherence, resource deficiencies, unacceptable harms or large benefits, or emerging information that makes the trial irrelevant, unnecessary, or unethical. If a trial stops early for apparent benefit, the estimates of treatment effect are more likely to be biased and prone to exaggeration [57,58].

### Numbers Analysed Item: Number of participants analysed in each group.

#### Example.

“… 300 were included in the analysis of the primary outcome (100 in the acetaminophen group, 100 in the ibuprofen group, and 100 in the codeine group)” [50].

#### Explanation.

Authors should report the number of participants included in the analysis for each intervention group. These data permit an assessment of whether participants were analysed according to their original group assignment, which is important, because failure to include all participants in the analysis may bias the results of the trial [22].

Several studies have reported deficiencies in journal and conference abstracts in reporting the number of participants included in the analysis [6–8,13,48,59]. In a review of RCTs in acute brain injury reported in journal abstracts, only 43% reported the number of participants included in the analysis [48]. In another evaluation of trials reported in abstracts for an oncology conference, only 40% reported the number of participants analysed, and only 6% indicated intention to treat analysis [8].

### Outcome Item: For the primary outcome, a result for each group and the estimated effect size and its precision.

#### Example.

“Treatment was successful for 682 (88%) of 778 patients recruited in the intervention group, and for 563 (76%) of 744 patients recruited in the control group (adjusted risk ratio [RR], 1.18; 95% confidence interval [CI], 1.03–1.34)” [33].

#### Explanation.

For the primary outcome, authors should report trial results as a summary of the outcome in each group (e.g., the number of participants with or without the event, or the mean and standard deviation of measurements), together with the contrast between groups known as the effect size. For binary outcomes, the effect size could be the relative risk, relative risk reduction, odds ratio, or risk difference. For survival time data, the measurement could be the hazard ratio or difference in median survival time. For continuous data, the effect measure is usually the difference in means. Authors should present confidence intervals for the contrast between groups and as a measure of the precision (uncertainty) of the estimate of the effect [22]. For abstracts not reporting the “primary” outcome of the trial (e.g., abstracts focusing on safety data or economic impacts), the secondary nature of the outcomes should be indicated, and, where possible, sufficient details of the primary outcome should be included to allow other findings to be taken in the proper context.

Several studies have observed deficiencies in the reporting of statistical results in journal abstracts [57,60–62]. For example, Pocock and colleagues [57] found that journal abstracts of RCTs tended to overemphasize statistically significant outcomes compared to the full journal article, leading to problems in interpretation of the results. Poor reporting of results is also a problem for trials presented in conference abstracts [7,8,59]. A study of 494 reports of RCTs in oncology found that only 26% of conference abstracts reported the size of the effect and significance of the result [7].

### Harms Item: Important adverse events or side effects.

#### Example.

“Adverse events were more common with topiramate vs placebo, respectively, including paresthesia (50.8% vs 10.6%), taste perversion (23.0% vs 4.8%), anorexia (19.7% vs 6.9%), and difficulty with concentration (14.8% vs 3.2%)” [63].

#### Explanation.

Most interventions have unintended and often undesirable effects as well as intended and beneficial effects. In order to make rational and balanced decisions, readers need information about the relative benefits and harms of an intervention. Authors should describe any important adverse (or unexpected) effects of an intervention in the abstract. If no important adverse events have occurred, the authors should state this explicitly [20].

Explicit reference to the reporting of harms in the title or abstract is also important for appropriate database indexing and information retrieval. Derry and colleagues [64] found that only 66 of 107 RCTs that reported data on adverse events in the full publication mentioned harms in the title or abstract; thus, harms could not have been identified for many of the articles in a search of titles and abstracts in an electronic bibliographic database.

Harms are also poorly reported in conference abstracts. A recent examination of over 800 ophthalmology conference abstracts reporting trials found that the majority (71%) did not report harms related to the treatment intervention, and harms were reported as a primary outcome measure in only 6% of abstracts [9].

### CONCLUSIONS Item: General interpretation of the results.

#### Example.

“Multivitamin supplementation reduced the incidence of low birth weight and small-for-gestational-age births but had no significant effects on prematurity or fetal death” [65].

#### Explanation.

The conclusions of the trial, consistent with the results reported in the abstract, should be clearly stated along with their clinical application (avoiding over-generalisation). Authors should balance the benefits and harms in their conclusions. Where applicable, authors should also note whether additional studies are required before the results are used in clinical settings [26].

### TRIAL REGISTRATION Item: Registration number and name of trial register.

#### Example.

“Trial Registry: www.clinicaltrials.gov; Identifier: NCT00412009” [33].

#### Explanation.

Nonpublication of entire trials and selective reporting of outcomes within trials has been well-documented [39,41,66]. Covert redundant publication can also cause problems in systematic reviews when results from the same trial are inadvertently included more than once [67]. To minimize or avoid these problems there have been many calls for trial registration [68]. Due to more recent serious problems of withholding data [69] there has been a renewed effort to register RCTs. By registering a RCT, authors typically report a minimal set of information and obtain a unique trial registration number.

In September 2004 the International Committee of Medical Journal Editors (ICMJE) indicated a change in their policy for publishing RCTs, saying that they would consider trials for publication only if they had been registered before the enrolment of the first patient (as of 1 July 2005) [70]. This position has resulted in a dramatic increase in the number of trials being registered [71].

In an abstract reporting a trial, authors should provide details of the trial registration number and name of trial register. Registration information will be particularly important for abstracts reported in conference meetings, as not all of them are subsequently published [1]. Such trial registration provides readers with a way to obtain more information about the trial and its results. Registration information will also help to link abstracts with subsequent full publications (or multiple abstracts from the same trial) and thus reduce the risk of inadvertent double-counting in systematic reviews.

### FUNDING Item: Source of funding.

#### Example.

“Funded by The Breast Cancer Research Foundation” [72].

#### Explanation.

Authors should report the source of funding for the trial as this is important information for readers assessing a trial. A recent systematic review showed that studies funded by the pharmaceutical industry had four times (odds ratio 4.05; 95% confidence interval 2.98–5.51) the odds of having outcomes favouring the sponsor than studies funded by other sources [73]. Similarly, authors should report any other sources of support, such as in the preparation of the abstract, presentation, or manuscript [74].

## Discussion

CONSORT for Abstracts strongly recommends the use of structured abstracts for reporting RCTs [75]; the full CONSORT Statement also supports their use [22]. Since 1987 when the Ad Hoc Working Group for Critical Appraisal of the Medical Literature [25–27] first published recommendations for the adoption of structured abstracts, many journals have promoted their use, and many different formats for structured abstracts now exist. We recognise that journals may have developed their own set of headings for abstracts [76,77]. It is not the intention of this reporting guide to suggest changes to these headings but to recommend what information should be reported within them when describing a RCT.

It is important to note that, because of the space limitations of an abstract, it will only ever be possible to provide limited information about a trial report. CONSORT for Abstracts sets out to recommend what information should be reported within these constraints when describing a RCT. Readers of abstracts should always try to obtain more information about a trial and its results, either by accessing the full publication or, in the case of unpublished conference abstracts [1], by contacting the authors for more information.

With the aim of greatly improving access to information about clinical trials and their results, the World Health Organization (WHO) recently established an International Clinical Trials Registry Platform. Their goal is to produce a single minimum standard for information that trialists should disclose before the trial begins [78,79]. Moreover, as registration of the trial methods has become more common, several forces have begun to advocate for the disclosure of trial results in specially designed repositories linked to trial registers. In June 2007, endorsing the WHO's International Clinical Trials Registry Platform, the ICMJE published an editorial recommending a standard abstract format for reporting results. The ICMJE suggested that CONSORT for Abstracts may be one such option [80]. At present, there is no formal consensus on international norms and standards for results reporting. The WHO International Clinical Trials Registry Platform has therefore established a Study Group on the Reporting of Findings of Clinical Trials to advise the WHO Registry Platform on matters related to the reporting of the findings of clinical trials. Full transparency and accountability require that all results of all trials are made available to the public in a timely manner.

Like the CONSORT Statement, CONSORT for Abstracts has been developed primarily for reporting the main results of parallel group RCTs (i.e., those relating to the prespecified primary outcome). There may well be instances where different types of trial information, such as composite outcomes, or different designs, such as cluster trials or noninferiority and equivalence trials, will require additional information not covered in this explanation and elaboration document. Possible additional abstract extensions may be warranted, as has been done for the CONSORT Statement for full reports [35,81].

The length of an abstract reporting a RCT using the CONSORT for Abstracts checklist is difficult to estimate. In developing the checklist 250 to 300 words were found to be sufficient to address all of the items in the checklist. Worked examples of using the CONSORT for Abstracts checklist are available on the CONSORT website at http://www.consort-statement.org/. In the past, MEDLINE has truncated journal abstracts at 250 words [82]. This has resulted in many journals setting word limits for abstracts in their journals at 250 words. However, since 2000 the National Library of Medicine increased the word limit for an abstract appearing in MEDLINE to 10,000 characters, which equates to over 1,000 words. While most abstract reports will not require anywhere near 1,000 words, such a word length will be sufficient to report even the most complex of trials in abstract form [82].

Clear, transparent, and accurate reporting of research is important because it enables readers to understand what was done and hence to evaluate the reliability and relevance of the findings. This extension of the CONSORT Statement aims to improve the reporting of RCTs in both the abstracts of journal articles and conference proceedings [83]. When using the CONSORT for Abstracts checklist we encourage authors to use it in conjunction with this explanation and elaboration document. We encourage journals and conference organisers to endorse the use of CONSORT for Abstracts by modifying their “Instructions to Authors” and drawing their readers' attention to this reporting guidance, perhaps through an editorial or by including a link to the checklist on the conference website. The most important benefit will be to enable readers to use abstracts more effectively and to assess the validity of the research more precisely. When key aspects of study methods are omitted, reader assessments are less certain, and might well take longer to make.

## Supporting Information

Text S1 Results of the Delphi Consensus Survey (69 KB RTF)

## Abbreviations

CONSORT: Consolidated Standards of Reporting Trials
CSE: Council of Science Editors
ICMJE: International Committee of Medical Journal Editors
STARD: Standards for Reporting Diagnostic Accuracy
WAME: World Association of Medical Editors
