# Supplementary material for: CONSORT for Reporting Randomized Controlled Trials in Journal and Conference Abstracts: Explanation and Elaboration
Source: PLoS Med. 2008 Jan 22;5(1):e20. doi: 10.1371/journal.pmed.0050020 (PMC2211558; doi:10.1371/journal.pmed.0050020)
Supplement: Supplementary file 1 [file pmed.0050020.sd001.rtf]

Text S1: Results of the Delphi consensus survey

We used a modified Delphi consensus method [Hasson F, Keeney S, McKenna H (2000) Research guidelines for the Delphi survey method. J Adv Nurs 32: 1008-1015] to select and reduce the number of possible checklist items; three Delphi rounds were used. A total of 109 participants, who were known to have an interest in the reporting of randomized trials, the structure of abstracts, or both were invited (by e-mail) to participate in an electronic survey and rate the importance of suggested checklist items. The electronic survey was initially piloted by members of the CONSORT for Abstracts Steering Committee. 

Invited survey participants included members of the CONSORT Group, the EQUATOR (Enhancing the QUAlity and Transparency Of Research) Network, journal editors, scientific programme committee chairs from several large international conferences (in 2006) and other researchers who have produced similar guidelines or research in this area. After participants were invited to join they were sent a copy of the survey, including instructions on how to complete it, along with a deadline for returning the completed forms. Participants were sent an e-mail reminder 48 hours prior to the completion deadline for each round of the survey. No other reminders were given. Participant responses were summarized and reported to back to participants at the end of each round of the survey.

Items included in the Delphi survey were arranged under headings which correspond to the conventional ordering of sections in a scientific article (i.e. title, authors, objectives, methods, results and conclusions). Each item was listed with a concise definition of what was meant, for example “Participants - Eligibility criteria for participants and the settings where the data were collected”.

Of the 106 participants invited to participate in the survey, 63 (61%) responded; three people declined citing lack of time (1), conflict of interest (1), disagreement with the purpose of the survey (1). 

Round One
Sixty participants completed the first round of the survey. Characteristics of the experts who agreed to participate are described in Table S1, below. Respondents included journal editors (13%), healthcare professionals (22%), methodologists (40%), statisticians (5%), trialists (7%) and other individuals with expertise in the reporting of randomized trials (13%).

In round one of the survey, participants were asked their views on the relative importance of the items on the checklist. They were asked to score each item on a 10-point Likert scale (ranging from 1, not important; to 10, very important). The mean score was then calculated for each item based on the participants' responses.

Participants also had the opportunity to comment on the checklist items or to suggest additional items. Content analysis was used to look through the comments made by participants in round one, to characterise them under different headings and to look for common themes to generate additional checklist items. Two themes came through quite strongly, the first was 'source of funding' and the second 'trial design (e.g. cluster, parallel, factorial)'. These two items were added as new items to round two of the checklist. 

Round two
Fifty-four participants completed the second round of the survey (90% of the participants from round one). Round two included all of the items from round one re-grouped in order of importance, using the mean scores (for each checklist item) from round one. 

Checklist items with a mean score of ≥ 8 were grouped as 'Included' checklist items. Items with a mean score of 6 or 7 were grouped as 'Possible' checklist items. Participants were told that these items had been ranked as of moderate importance and might not be included in the final checklist. Items with a mean score of 1 to 5 were grouped as 'Rejected' checklist list items. Participants were told that these items had been ranked as of low importance and would not be included in the final checklist unless they received much higher scores in round two. As noted above, two additional items were included in the checklist in this round.

The participants were asked again to score the relative importance of each checklist item using the same scoring system. The mean score was then calculated for each checklist item based on the responses in round two.

Round three
Forty-seven participants completed the third round of the survey (88% of the participants from round two). Round three included only those items ranked as of high importance by survey participants in round one and two (i.e. those items with a mean score of ≥ 8). Items repeatedly scored as of low importance (i.e. those with a mean score of 1 to 5) were removed from the checklist (Table S2, below). 

However, because there was still uncertainty following round two regarding those items scored as of moderate importance (i.e. those with a mean score of 6 or 7), participants in round three were asked specifically for their views on whether these items should be included, excluded or optional in the final checklist. These items included randomization generation (description of the method used to generate the random allocation sequence), allocation concealment (description of the method used to keep the sequence hidden from those allocating the intervention to participants until the point of assignment) and source of trial funding. The responses are given in Table S3, below. Source of funding was seen as an important item which should be included in the checklist. There was less certainty surrounding the method of allocation concealment and randomization generation. These two items were discussed at length at the subsequent meeting of the CONSORT Group in Montebello, Canada, where the consensus was that details of randomization i.e. how participants were allocated to interventions, should be included in the final checklist.

Table S1: Profile of experts who participated in the first Delphi round

	n = 60 (%)	
Male	33 (55)	
Female	27 (45)	
Age		
   < 35 years	6 (10)	
   35 – 44	25 (42)	
   45 – 54	14 (23)	
   55 - 64	10 (17)	
   > 65 years	5 (8)	
Editor	8 (13)	
Healthcare professional	13 (22)	
Methodologist	24 (40)	
Other	8 (13)	
Statistician	3 (5)	
Trialist	4 (7)	


Table S2: Items rejected after round two of the Delphi survey

Item	Description	Mean score R1	Mean score R2	
Background	Scientific background and explanation of rationale	5	5	
Trial date	Accrual date of the trial (i.e. date of first or last randomization, or both)	5	5	
Sample size calculation	Method of determining the sample size	4	4	
Statistical methods	Description of statistical methods to compare groups for the primary outcome	5	5	
Randomization implementation	Information on who generated the allocation sequence, who enrolled the participants, and who assigned participants to their groups	4	4	
Baseline data	Baseline demographic and clinical characteristics of each group	5	5	
Generalizability	Discussion of generalizability (external validity) of the trial findings	5	4	


Table S3: Delphi round three responses for possible checklist items (mean score 6 or 7 in round two)

Item	Description	Include	Exclude	Optional	
Randomization generation	Description of the method used to generate the random allocation sequence	8 (17%)	20 (43%)	19 
(40%)	
Allocation concealment	Description of the method used to keep the sequence hidden from those allocating the intervention to participants until the point of assignment)	14 (30%)	13 (28%)	20 
(42%)	
Funding	Source of trial funding	31 (66%)	4
(9%)	12 
(25%)
